# Effect of Thermomagnetic Treatment on Structure and Properties of Cu–Al–Mn Alloy

**DOI:** 10.1186/s11671-017-2052-6

**Published:** 2017-04-20

**Authors:** A. N. Titenko, L. D. Demchenko, A. O. Perekos, O. Yu Gerasimov

**Affiliations:** 1 Institute of Magnetism of the National Academy of Sciences and Ministry of Education and Science of Ukraine, 36-b Akademika Vernadskoho Blvd, Kyiv, 03142 Ukraine; 2 National Technical University of Ukraine “Kyiv Polytechnic Institute”, 37 Peremohy Ave, Kyiv, 03056 Ukraine; 3 G.V. Kurdyumov Institute for Metal Physics of the National Academy of Sciences of Ukraine, 36 Akademika Vernadskoho Blvd, 03680 Kyiv, Ukraine

**Keywords:** Nanoparticles, Magnetization, Magnetic susceptibility, Magnetic field, Microhardness

## Abstract

The paper studies the influence of magnetic field on magnetic and mechanical properties of Cu–Mn–Al alloy under annealing. The comparative analysis of the magnetic field orientation impact on solid solution decomposition processes in a fixed annealing procedure is held using the methods of low-field magnetic susceptibility, specific magnetization, and microhardness test. The paper highlights changes in the magnetic and mechanical properties of Cu–Al–Mn alloy as the result of change in a critical size of forming precipitated ferromagnetic phase and determines correlation in the behavior of magnetic and mechanical properties of the alloy, depending on a critical nucleus size of forming precipitated ferromagnetic phase.

## Background

In the market of new materials, the functional materials having unusual properties are in great demand, among which the ferromagnetic shape memory alloys are predominant. The control over such properties is exercised using force, thermal, and magnetic fields. An important aspect of improvement in the material properties is to create a nanostructured state, which has significant advantages in magnetic and mechanical characteristics in contrast to the bulk materials in crystalline or amorphous state. Magnetization and magnetic anisotropy in case of nanoparticles can be significantly greater than that of a bulk sample, and a difference between the Curie temperature (*T*_c_) reaches hundreds of degrees [1–3]. Magnetic nanomaterials have a number of unusual properties, in particular, giant magnetoresistance, abnormally large magnetocaloric effect, and others [4].

Cu–Al–Mn alloys are one of the most interesting ferromagnetics with shape memory (SM). They demonstrate an unusual magnetic behavior, in particular, superparamagnetism [5] and giant magnetoresistance [6], and specific mechanical properties such as SM effect, thermoelasticity, superelasticity, and plasticity of transformation [7, 8]. Moreover, the Cu–Al–Mn alloys exhibit a superelastic strain of about 7%, which is comparable to that of Ti–Ni alloys [9, 10].

It was reported [9, 11, 12] that polycrystalline Cu–Al–Mn shape memory alloys with a low Al content between 16 and 18 at.% have a good balance between workability and SM properties. These alloys demonstrate excellent ductility and exhibit SM properties such as superelasticity and the one- or two-way memory effect based on martensitic transformation. The β (L2_1_) phase significantly extends to the low Al content region by the addition of Mn [10]. The degree of order in the phase is lowered by decreasing the Al content. Doping of binary Cu–Al alloys with Mn slows the decomposition of solid solution under aging and alters the physical and mechanical characteristics of the material. Thus, Cu-based alloys with 14 wt% Al and 3 wt% Mn and with 13 wt% Al and 7.5 wt% Mn have the same start temperature of martensitic transformation (M_s_ ≈ −80 °C); however, the hardness of the matrix differs by 50% (300 and 200 HV) [13].

To get optimal properties, these alloys usually undergo an additional thermal, mechanical, or magnetic treatment. Thus, aging of Cu–Al–Mn alloys leads to the formation of a system of nanoscale particles of ferromagnetic Cu_2_MnAl phase in a paramagnetic Cu_3_Al matrix [5], and annealing in magnetic field increases the *T*_c_ of Cu–Al–Mn alloys [14]. At the same time, the heat treatment allows to control number and size of particles in the alloy and also the martensitic transformation temperature and hysteresis, which depend on characteristics of precipitated particles [15, 16]. Thus, the clarification of the possibility to control the magnetic and mechanical characteristics of Cu–Al–Mn alloys under annealing in magnetic field is of particular interest.

The purpose of this paper was to study the effect of preliminary thermomagnetic treatment (TMT) on structure, magnetic, and mechanical properties of Cu–Al–Mn alloy under the formation of the system of nanoscale magnetic particles.

## Methods

The object of investigation was the alloy of the following composition: 84.7Cu-11.4Al-3.9Mn (% by weight), smelted in an induction furnace in an argon atmosphere. Samples were prepared as rods of the length of 15 mm and the cross section of 3.5×3.5 mm. After the homogenizing annealing at 1123 K for 10 h, the alloy samples were quenched in water and then annealed in air at 473 K for 3 h in magnetic field with intensity of 1.5 kOe having different orientation (perpendicular or parallel to the main axis of sample) or without field. Magnetization was measured with a ballistic magnetometer, electrical resistance was determined using a four-point method, and low-field magnetic susceptibility was studied using an induction method. The phase composition of samples was examined using an X-ray diffractometer Rigaku Ultima IV in K_α_-monochromatic radiation of Cu anode. The chemical composition of the alloy was defined according to the data of energy dispersive X-ray fluorescence analysis accurately to within ±0.5%. The microstructure of alloy was studied using a scanning electron microscope (SEM). A size of precipitated nanoparticles was estimated by the two-pass method of atomic force microscopy (AFM) using a scanning probe microscope (SPM) Solver PRO-M with a magnetic cobalt probe NSG01/Co with the size of 130 × 35 × 2 μm and the distance from the probe to the surface for the second pass of 100 nm.

## Results and Discussion

**Fig. 1:**
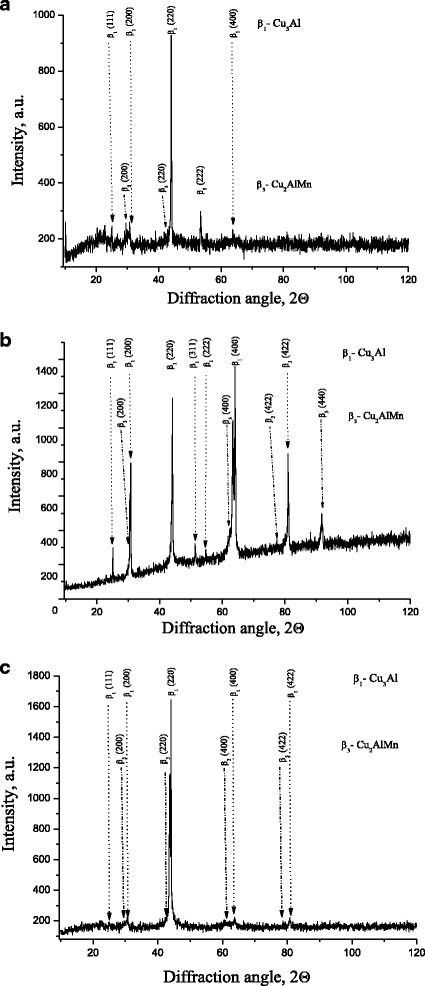
X-ray diffraction patterns of Cu–Al–Mn alloy samples annealed at 200 °C for 3 h without magnetic field (**a**), in magnetic field perpendicular to the main axis of a sample (**b**), and in magnetic field parallel to the main axis of a sample (**c**)

The results of X-ray phase analysis (Fig. 1) reveal that the high-temperature β_1_ phase (with ordered fcc structure of DO_3_ type and a composition close to Cu_3_Al) is formed under cooling by quenching in water as a result of ordering of the high-temperature β phase with a bcc lattice of A2 type [17]. The subsequent annealing (for aging) of the high-temperature β_1_ phase results in the precipitation of ferromagnetic ordered β_3_ phase (with L2_1_-type structure and a composition close to Cu_2_AlMn) in the paramagnetic β_1_ matrix [18, 19], as evidenced by an appearance of weak intensity reflections from β_3_ phase at appropriate diffraction angles.

The weak intensity of reflections can indicate that the particles of precipitated phase are high-dispersed or nanosized. In its turn, the change in orientation of samples relative to the magnetic field direction (perpendicular or parallel) affects quantity and size of precipitated particles, which is manifested in the redistribution of the intensities of diffraction peaks.

**Fig. 2:**
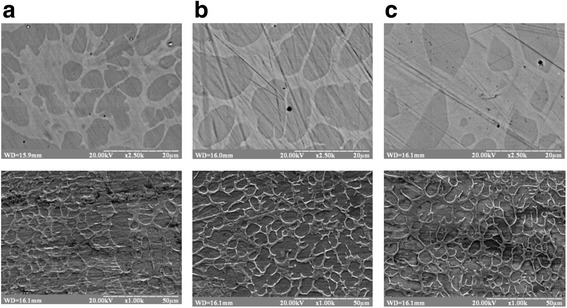
The microstructures of alloy samples annealed at 200 °C for 3 h without magnetic field (**a**), in perpendicular magnetic field (**b**), and in parallel magnetic field (**c**)

Figure 2 shows the microstructure of the alloy with different regimes of TMT obtained using SEM. The scanning electron micrographs indicate the existence of two-phase region, consisting of two β_1_-solid solutions with different concentration of elements. In the X-ray diffraction patterns, the split Bragg lines of β_1_ phase are observed that can testify to the solid solution decomposition into two phases of the same structure, but different concentration of Al and Mn. So, the dark component (etched stronger) on the microstructures belongs to the β_1_ phase with a higher amount of Al (12.5 ÷ 15.4 wt.%) and a lower content of Mn (3 ÷ 5.4 wt.%) in contrast to the lighter component with the concentration of Al (8.3 ÷ 9.9 wt.%) and Mn (8.7 ÷ 10.5 wt.%).

It is assumed that during the annealing of aging alloys, the magnetic field lines up the precipitated anisotropic ferromagnetic (β_3_-Cu_2_AlMn) particles elongated in the field direction in regular-oriented chains. Unfortunately, since the aging time was short and, as a consequence, a low volume fraction of particles has been precipitated during the initial stages of the solid solution decay, such kind of particle orientation along the field was not observed when using X-ray and SEM analyses. However, there are some changes in the magnetic parameters of the alloy after TMT, contrary to the alloy annealed without field.

To confirm the presence of nanoparticles on the sample surface of the Cu–Al–Mn alloy, the two-pass method of AFM was used. The first pass was applied for the pre-measurement of a sample surface profile, and the second pass was performed in the phase image mode to measure phase shift distribution on the sample surface, which reflects distribution of the material characteristics.

**Fig. 3:**
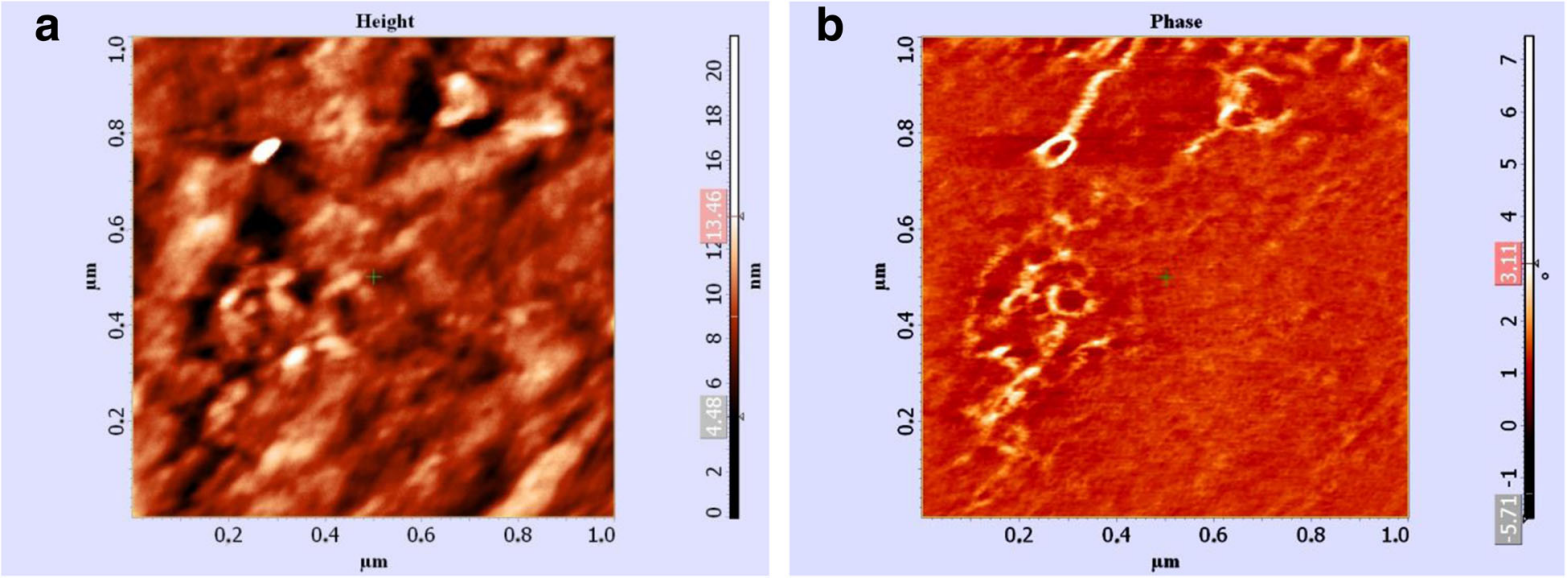
The microstructures of Cu–Al–Mn alloy surface: a surface image in micron scale (**a**) and the same image in phase contrast (**b**)

The image of the sample surface in phase contrast was received (Fig. 3). If the surface of sample is inhomogeneous by its properties, the appropriate phase shift is observed. The size of nanoparticles is in the range of 20–40 nm.

**Fig. 4:**
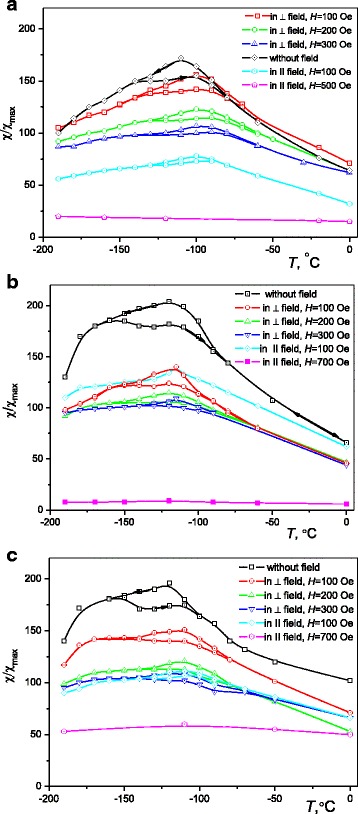
The temperature dependence of low-field magnetic susceptibility (*χ*/*χ*_max_), registered under different regimes, for Cu–Al–Mn alloy samples annealed at 200 °C for 3 h without magnetic field (**a**), in perpendicular magnetic field (**b**), and in parallel magnetic field (**c**). *Arrows* indicate heating–cooling

**Fig. 5:**
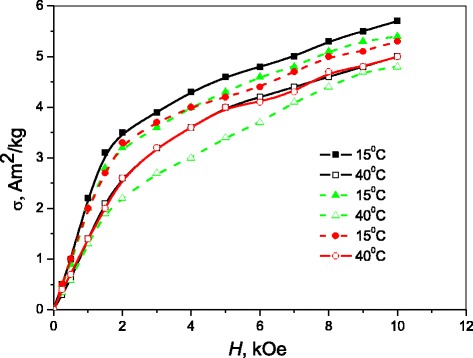
The field dependences of specific magnetization, registered at 15 and 40 °C, for Cu–Al–Mn alloy samples annealed at 200 °C for 3 h without magnetic field (*black*), in perpendicular magnetic field (*red*), and in parallel magnetic field (*green*)

The behavior of specific magnetization *σ* and low-field magnetic susceptibility *χ* indicates a difference in the magnetic characteristics of samples depending on their TMT (Figs. 4 and 5). The values of these characteristics tend to vary as a result of changes in size of precipitated ferromagnetic (β_3_-Cu_2_AlMn) particles which define magnetic properties of the alloy under TMT.

According to the low-field magnetic susceptibility temperature dependences (Fig. 4), the signal increases as a result of paramagnetic ordering (PMO) and then falls as a result of transition to spin glass (SG) type state, and the transition temperature *T*_*B*_ corresponds to the blocking temperature of magnetic moments [5]. The formation of such state is determined by the indirect exchange of RKKY type. To rotate the magnetic moment of the ferromagnetic (β_3_-Cu_2_AlMn) particle along the magnetic field, it is required to overcome the energy barrier:

1 $$ \varDelta E\approx {K}_V V $$

where *K*_*V*_ is an effective constant of magnetic anisotropy and *V* is a volume of the particle. In the absence of magnetic field, the temperature of PMO-SG transition (*T*_*B*_) is defined as:

2 $$ {T}_B\approx {K}_V V/25.3{k}_B $$

In our case, *T*_*B*_ is of a larger magnitude for the treatment without magnetic field in consequence of a larger size of precipitated particles, since the external magnetic field causes the reduction of a critical radius (*R*_*c*_) of the precipitated particles [20]:

3 $$ {R}_c={\gamma}_s/\left(\mu H+{g}_v-{e}_v\right) $$

where *g*_*v*_ is a specific free energy of chemical nature, *γ*_*s*_ is a surface energy per unit area, *e*_*v*_ is a specific elastic energy, *μ* is a magnetic moment of the ferromagnetic phase, and *H* is a magnetic field intensity.

According to (2), the shift of *T*_*B*_ on the *χ*/*χ*_max_ *(T)* curves towards lower temperatures under effect of TMT can be caused by a decrease in volume of the precipitated ferromagnetic particle. At the same time, the shift of *T*_*B*_ towards high temperatures for the same TMT regimes under applied magnetic field of different intensity (100 ÷ 700 Oe) can be caused by the strong field dependence of *K*_*V*_, although the magnitude of magnetic field has no effect on the transition temperature (*T*_*B*_).

As it is shown on *σ(H)* curves (Fig. 5), the alloy magnetization at higher temperatures (at 40 °C) exhibits a characteristic superparamagnetic behavior. A theory of superparamagnetism [21] provides for the relationship *μ*_*ef*_ *H*_*s*_ ≈ *k*_*B*_ *T*. The magnetization *σ(H)* for the samples aged without field is of a larger magnitude as compared to the sample aged in magnetic field (Fig. 5).

A size of the nanoparticles can be estimated by the slope of the linear section of *σ(H)* dependence at *H →* 0, which in the case of temperature *T* = 40 °C has a smaller slope and a smaller magnetization magnitude that also points to reduction of a size of the particles when the aging occurs in magnetic field.

**Fig. 6:**
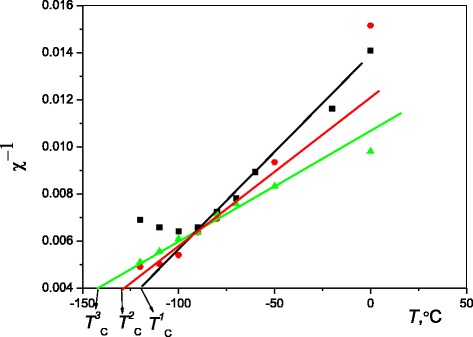
The temperature dependences of the inverse of magnetic susceptibility (χ^−1^) of Cu–Al–Mn alloy samples annealed at 200 °C for 3 h without magnetic field (*black*), in perpendicular magnetic field (*red*), and in parallel magnetic field (*green*)

The paramagnetic Curie temperature (Fig. 6) is significantly higher in samples annealed without magnetic field (${T}_{\mathrm{C}}^1$) as compared to samples annealed in perpendicular (${T}_{\mathrm{C}}^2$) or parallel (${T}_{\mathrm{C}}^3$) field that testifies to a bigger volume fraction of particles in samples aged without field.

An increase in microhardness of the samples annealed in magnetic field (Table 1) can also testify to a decrease in size of precipitated nanoparticles during TMT. The microhardness of samples increases under annealing in magnetic field, and the maximum value of microhardness (4.8 GPa) is achieved after TMT in parallel field, in contrast to a sample having 4.4 GPa microhardness after annealing without magnetic field.

**Table 1:** Microhardness of alloys after different TMT

Treatment (annealing at 200 °C for 3 h)	Average microhardness *H*_*μ(*ave)_ (GPa)
Without magnetic field	4.4
In magnetic field perpendicular to the sample axis	4.6
In magnetic field parallel to the sample axis	4.8

No anomalies were observed on the temperature curves of electrical resistivity of the investigated samples.

## Conclusions

This paper studies the effect of thermomagnetic treatment of 84.7Cu-11.4Al-3.9Mn (wt.%) alloy on the structure, magnetic, and mechanical properties. The correlation between the magnetic and mechanical behavior of the investigated alloy in terms of the morphology of nanoparticle formation of ferromagnetic precipitated phase at 200 °C for 3 h both in magnetic field having different orientation, and without it, was found, and the magnetic field dependence on a critical size of the precipitated phase was shown. The treatment in parallel magnetic field causes the reduction in a size of nanoparticles that appears in the decrease of magnetic characteristics of the alloy and in the increase of the alloy microhardness.

## Abbreviations

AFM: Atomic force microscopy
PMO: Paramagnetic ordering
SEM: Scanning electron microscope
SG: Spin glass
SM: Shape memory
SPM: Scanning probe microscope
TMT: Thermomagnetic treatment
